# Behavioral Characterization of *GCLM*-Knockout Mice, a Model for Enhanced Susceptibility to Oxidative Stress

**DOI:** 10.1155/2011/157687

**Published:** 2011-04-27

**Authors:** Toby B. Cole, Gennaro Giordano, Aila L. Co, Isaac Mohar, Terrance J. Kavanagh, Lucio G. Costa

**Affiliations:** ^1^Department of Environmental and Occupational Health Sciences, University of Washington, Seattle, WA 98195, USA; ^2^Division of Medical Genetics, University of Washington, Seattle, WA 98195, USA; ^3^Department of Genome Sciences, University of Washington, Seattle, WA 98195, USA; ^4^Department of Human Anatomy, Pharmacology and Forensic Science, University of Parma Medical School, 43121 Parma, Italy

## Abstract

Glutathione (GSH) is a major player in cellular defense against oxidative stress. Deletion of the modifier subunit of glutamate cysteine ligase (*GCLM*), the first and the rate-limiting enzyme in the synthesis of GSH, leads to significantly lower GSH levels in all tissues including the brain. *GCLM*-knockout (*Gclm^−/−^*) mice may thus represent a model for compromised response to oxidative stress amenable to *in vitro* and *in vivo* investigations. In order to determine whether the diminished GSH content would by itself cause behavioral alterations, a series of behavioral tests were carried out comparing young adult *Gclm^−/−^* with wild-type mice. Tests included the rotarod, acoustic startle reflex and prepulse inhibition of the startle reflex, open field behavior, and the platform reversal variant of the Morris Water Maze. Results showed no differences between *Gclm^−/−^* and wild-type mice in any of the neurobehavioral tests. However, more subtle alterations, or changes which may appear as animals age, cannot be excluded.

## 1. Introduction

Oxidative stress refers to the cytotoxic consequences of reactive oxygen species (ROS), which are generated as byproducts of normal and aberrant metabolic processes that use molecular oxygen. The tripeptide glutathione (GSH; *γ*-glutamyl-cysteinyl-glycine) is one of the most abundant cellular thiols. GSH is a major player in cellular defense against ROS, because it nonenzymatically scavenges both singlet oxygen and hydroxyl radicals, and is used by glutathione peroxidases and glutathione transferases to limit the levels of certain reactive aldehydes and peroxides within the cell [1, 2]. When ROS production exceeds the antioxidant defense capacity of the cell, oxidative stress ensues, leading to the damage of DNA, proteins, and membrane lipids. The first and the rate-limiting step in the synthesis of GSH is carried out by glutamate-cysteine ligase (GCL; [1]). The enzyme consists of two subunits, a larger (73 kD) catalytic subunit (GCLC) and a smaller (31 kD) modifier, or regulatory, subunit (*GCLM*), which are coded by separate genes [3]. GCLC alone provides catalytic activity and is the site of GSH feedback inhibition. By lowering the *K*_*m*_ of GCL for glutamate and raising the *K*_*i*_ for GSH, *GCLM*, although enzymatically inactive, plays an important regulatory function, as the holoenzyme (GCLholo) has higher catalytic efficiency than GCLC [3, 4]. While disruption of the *Gclc* gene in mice is embryolethal [5], no overt phenotype is observed upon disruption of the *Gclm* gene in mice [4, 6, 7]. In the absence of *GCLM*, the ability of GCLC to synthesize GSH is drastically reduced [8]. In tissues from *Gclm^−/−^* mice, GSH levels are only 10–30% of those found in *Gclm^+/+^* animals [6, 7, 9]. In brain tissue and cells, GSH levels in *Gclm^−/−^* mice are 17–35% of those present in wild-type mice [6, 10, 11].

*Gclm^−/−^* mice are more sensitive to the hepatotoxicity of acetaminophen [7], and neurons and astrocytes isolated from the brain of *Gclm^−/−^* mice have been shown to be particularly susceptible to the toxicity of agents that increase oxidative stress, such as domoic acid [6], certain organophosphorus insecticides [12], methylmercury and PCBs [13], and polybrominated diphenyl ethers [10]. 

A relatively common C588T polymorphism has been discovered in the 5′-flanking region of the human *GCLM* gene [14]. Individuals carrying the T allele have a lower promoter activity in a luciferase reporter gene assay in response to oxidants and significantly lower plasma GSH levels [14]. These individuals are also at risk for myocardial infarction and present with impairments in nitric oxide-mediated coronary vasomotor function [14, 15]. An association between *GCLM* polymorphisms and schizophrenia has also been suggested [16] but is still controversial [17, 18].

Nevertheless, individuals with *GCLM* polymorphisms leading to lower GSH levels would be expected to display an enhanced sensitivity to the adverse effects of oxidative stress. The *Gclm^−/−^* mouse thus represents a useful model for such *GCLM* polymorphisms, amenable for *in vitro*, as well as *in vivo* studies. In order to extend *in vitro* observations to an *in vivo* situation, an initial behavioral characterization of *Gclm^−/−^* mice was carried out, to determine whether the genetically determined diminished GSH level would by itself affect behavioral outcomes. Indeed, glutathione dysregulation is associated with the etiology and progression of several diseases, including neurotoxic and neurodegenerative disorders [19, 20]. 

## 2. Materials and Methods

### 2.1. Generation of Gclm-Null Mice and Genotyping

All animal use protocols were approved by the Institutional Animal Care and Use Committee at the University of Washington, and experiments were carried out in accordance with the National Research Council Guide for the Care and Use of Laboratory Animals, as adopted by the National Institutes of Health. Wild-type and *Gclm*-null (*Gclm^−/−^*) mice of backcrossed C57Bl/6J (B6.129) strain background [6, 7] were housed in a centralized, AAALAC-accredited, and specific pathogen-free facility at the University of Washington. Mice were maintained in a 12 h light-dark cycle with ad libitum access to food (standard mouse chow) and water. Male and female mice hemizygous for the *Gclm* deletion (*Gclm*-Hz) were intercrossed, generating wild-type, *Gclm^−/−^*, and *Gclm*-Hz mice, in the expected Mendelian ratios [21].

To genotype pups, genomic DNA was isolated from ear punch tissue using a Qiagen DNeasy kit, and mice were genotyped by PCR amplification of the wild-type and disrupted *Gclm* alleles (i.e., amplification of *β*-geo), as previously described [6, 7].

As seen previously, all pups developed normally and exhibited no differences in phenotypic landmarks compared to wild-type littermates. At weaning, mice were transferred to the neurobehavioral testing facility and housed two to four per cage for the duration of testing.

### 2.2. Neurobehavioral Assessment

One wild-type (total = 12) or *Gclm^−/−^* (total = 13) male mouse, each taken from a different litter, was used for neurobehavioral testing. Tests were chosen to investigate possible differences between the two mouse genotypes in sensory functions, motor activity and coordination, and learning and memory.

Auditory startle and prepulse inhibition of startle were tested at 12 weeks of age using an automated auditory startle chamber (San Diego Instruments). During a 15-minute test session, mice were placed in the startle chamber and presented with 30 stimuli at randomized intervals. The stimuli consisted of a 120 dB tone, a 120 dB tone preceded by a 70 dB prepulse, or a “null” stimulus involving no tone. Each type of stimulus was presented 10 times. The order of stimulus presentation was first determined using a random number table, after which each mouse received the stimuli in the same order. The startle chambers used a piezoelectric sensor to measure the maximum amplitude (*V*_max_) of the startle response after each stimulus and the latency to the maximum startle response (*T*_max_). Prepulse inhibition of startle was calculated as the percent inhibition of the auditory startle response by the 70 dB prepulse, after subtracting the startle response to the null stimulus [22, 23]. 

A rotarod (Coulbourn Instruments) was used to test motor coordination and cerebellar learning [24, 25] at 13 weeks of age. Mice were placed on the rotarod cylinder, which accelerated to 5 rpm/min from a baseline rate of 3 rpm. Latency to fall off the cylinder was recorded for each of four successive trials, with a 5 min intertrial interval.

Open-field behavior and locomotor activity were tested at 20 weeks of age using a Tru-Scan photo beam tracking system (Coulbourn Instruments, Whitehall PA). Mice were placed in an open-field arena that was 25.4 cm wide, 25.4 cm deep, and 40.64 cm high, and movements and behaviors were recorded for 15 min using dual sensor rings to measure infrared beam breaks in the horizontal or vertical plane. Beams were spaced 1.52 cm apart, providing 0.76 cm spatial resolution. Data were collected in 30 sec bins, and totals over the 15 min testing period were calculated as the sum of the 30 individual values. Data were also analyzed individually for the first 5 min period, the second 5-min period, and the third 5 min period. Specific measures included total number of movements, total movement time, total rest time, ambulatory move time, latency to first movement, latency to first ambulatory movement, total movement distance, ambulatory distance, mean velocity, ambulatory velocity, distance traveled in arena margin and arena center, time spent in arena margin and arena center, number of entries into arena center, time spent in back half and front half of arena, number of entries into back half and front half of arena, number of entries into vertical plane, time spent in vertical plane, number of jumps from floor plane, number of movements in vertical plane, number of stereotypic movements, number of stereotypic episodes, total time of stereotypic behavior, and number of counterclockwise and clockwise center point rotations. Data were collected automatically using Tru-Scan 2.0 software, and raw data were exported for analysis by Microsoft Excel.

A platform-reversal variant of the Morris water maze was used to test learning and memory [26–28] beginning at 14 weeks of age. This test utilized the polytrack system above. The maze consisted of a 165-gallon (624.6 liter), circular, galvanized stock tank, 4 ft (1.22 meter) in diameter and 2 ft (0.61 meter) in height, filled with room temperature water. A 10 cm square plexiglass stand was placed in the tank just below the water level to serve as the escape platform. A Polytrack system (San Diego Instruments) was used to track the location of the mice in the maze. Stationary objects surrounding the tank were used as spatial cues. Mice were trained for seven days, three trials per day, at 30 min intertrial intervals, to acquire the task. On the first trial, mice were dropped randomly at one of the four drop locations and allowed to explore the tank and become familiar with swimming. Mice were then guided to the escape platform and were held on the platform for 30 seconds, and then they were taken out of the tank, dried off, and placed under a heat lamp. On subsequent trials, mice were dropped into the tank and given 60 seconds to find the platform. Once the mice found and climbed onto the platform, the test was stopped and the latency to find the platform was recorded. After the 21-trial acquisition phase, the platform was moved to the opposite quadrant, and mice were tested for an additional 21 trials, with 3 trials per day separated by a 30 min intertrial interval. Latency to find the platform was measured as above. One month following the last reversal trial, mice were tested for retention using a probe test. The platform was removed, and mice were placed into the tank at a random drop location and allowed to swim for 2 minutes. Dwell time in each quadrant, average distance from target (previous location of platform), and number of target crossings were recorded. 

### 2.3. Statistical Analysis

Data were analyzed with Microsoft Excel. Differences between genotypes were tested for statistical significance by Student's *t*-test, followed in some cases by a Bonferroni correction for multiple testing. Results are reported as mean ± SE (*n* = 12-13).

## 3. Results

As previously reported [6, 7], *Gclm^−/−^* mice and wild-type mice were born in the expected Mendelian ratios and exhibited no obvious developmental differences during the postnatal developmental period. Adult male and female *Gclm^−/−^* mice had slightly lower bodyweights than wild-type mice (Table 1). Only male mice were used for behavioral testing.

**Figure 1:**
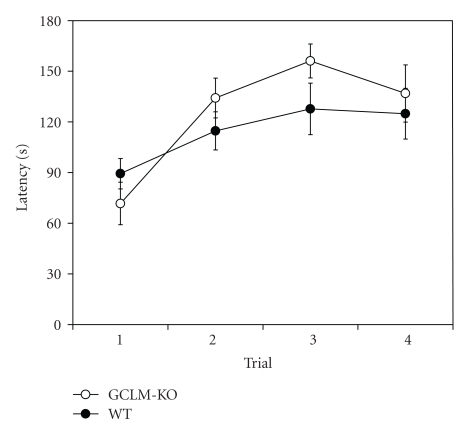
Rotarod. Mice were placed on a rotarod, and latency to fall from the rotarod was recorded for each of 4 successive trials. Trials were separated by a 1 min interval. Results are shown as mean ± SE (*n* = 12-13). WT: wild-type mice; *GCLM*-KO: *Gclm^−/−^* mice.

At 13 weeks of age, motor coordination and cerebellar learning were tested using a rotarod (Figure 1). Wild-type and *Gclm^−/−^* mice both learned the task, and latency to fall off the rotarod increased with each successive trial (trial 1 versus trials 2, 3, and 4: wild-type, *P* < .05; *Gclm^−/−^*, *P* < .01). There were no significant differences in latency between *Gclm^−/−^* and wild-type mice on trials one (*P* = .27), two (*P* = .24), three (*P* = .13), or four (*P* = .60), nor when averaging latencies across all four trials (*P* = .42). 

**Figure 2:**
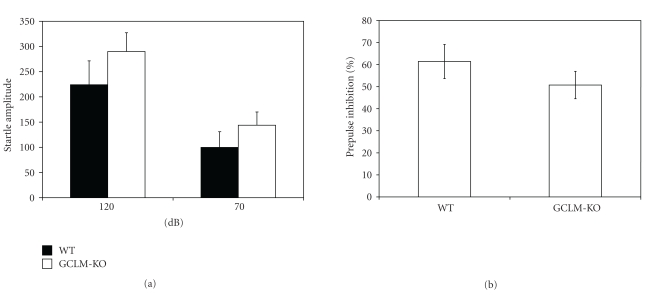
Prepulse inhibition/startle. Auditory startle response was measured using a startle chamber. Startle amplitude was similar in wild-type and *Gclm^−/−^* mice, and a 70 dB prepulse inhibited the subsequent startle response to a similar extent in wild-type and *Gclm^−/−^* mice. Results are shown as mean ± SE (*n* = 12-13). WT: wild-type mice; *GCLM*-KO: *Gclm^−/−^* mice.

Auditory startle and prepulse inhibition of startle were tested at 12 weeks of age (Figure 2). There were no differences in auditory startle amplitude (*P* = .27) between wild-type and *Gclm^−/−^* mice. When the 120 dB auditory stimulus was preceded by a 70 dB prepulse, the startle amplitude was significantly reduced (*P* < .05) in both wild-type and *Gclm^−/−^* mice (Figure 2). There were no differences between wild-type and *Gclm^−/−^* mice in the magnitude of this prepulse inhibition of startle (*P* = .28). Latencies to maximum startle were also similar in wild-type and *Gclm^−/−^* mice for both auditory startle (22.6 ± 1.8 msec and 20.8 ± 1.5 msec, resp.; *P* = .46) and prepulse inhibition of startle (31.9 ± 4.2 msec and 25.9 ± 1.8 msec, resp.; *P* = .18). 

**Figure 3:**
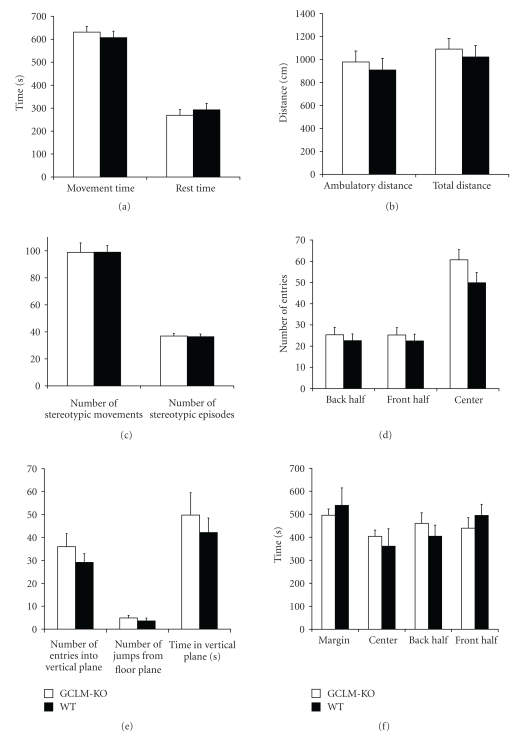
Open-field behavior. Mice were placed in an open-field chamber, and movements and behaviors were recorded for 15 min using infrared beam breaks in the horizontal or vertical plane. There were no differences between *Gclm^−/−^* and wild-type mice in ambulatory activity, time spent in different parts of the chamber, stereotypic movements, or vertical movements. Results are shown as mean ± SE (*n* = 12-13). WT: wild-type mice; *GCLM*-KO: *Gclm^−/−^* mice.

Locomotor activity and behavior in an open field were tested at 20 weeks of age, by placing the mice in a small arena and recording movements and behaviors for 15 min (Figure 3). *Gclm^−/−^* and wild-type mice were nearly identical in all measures (*P* > .18), including locomotor activity, total movement time, ambulatory distance traveled, time spent in different areas of the arena, number of stereotypic movements, and number of entries into the vertical plane (Figure 3).

**Figure 4:**
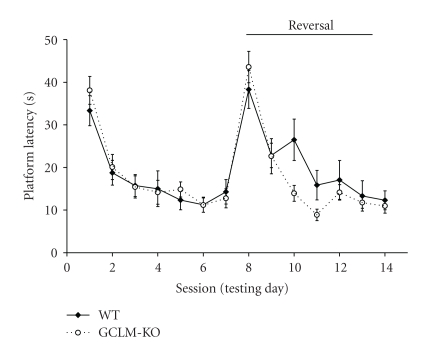
Morris water maze. Mice were tested in the Morris water maze for 14 days, 3 trials per day. During the acquisition phase (days 1–7), mice learned to find a submerged platform using spatial cues. On the 8th day, the platform was moved to the opposite quadrant and mice were tested for their ability to find the new platform location. There were no significant differences in acquisition or reversal between *Gclm^−/−^* and wild-type mice. Results are shown as mean ± SE (*n* = 12-13). WT: wild-type mice; *GCLM*-KO: *Gclm^−/−^* mice.

Learning and memory were tested using a platform-reversal variant of the Morris water maze, beginning at 14 weeks of age. *Gclm^−/−^* mice acquired the task equally as well as wild-type mice; latency to find the hidden submerged platform decreased (*P* < .001) over the seven daily testing sessions for both mouse genotypes (Figure 4). Platform reversal began on the eighth day, when the platform was moved to the opposite quadrant. Both wild-type and *Gclm^−/−^* mice had difficulty finding the new platform location on the first day, reflected in their increased latencies to find the platform in session eight but were able to learn the new platform location over subsequent training sessions (Figure 4; *P* < .0001). There were no significant differences between wild-type and *Gclm^−/−^* mice in either acquisition or reversal of the task (*P* > .24). While wild-type mice showed a tendency toward increased platform latency on session ten, when compared to *Gclm^−/−^* mice, this difference was not statistically significant (*P* = .24). One month after the end of the platform-reversal testing, retention was tested once for each mouse, using a probe test. Mice were placed in the Morris water maze and were allowed to search for two minutes in the absence of a platform. There were no differences between genotypes (*P* > .11) in percent dwell time in the platform quadrant (wild-type: 23.9 ± 1.9%; *Gclm^−/−^*: 19.7 ± 1.8%), percent dwell time in the reversal quadrant (wild-type: 27.8 ± 1.5%; *Gclm^−/−^*: 34.0 ± 3.4%), or in the average distance from target (wild-type: 38.7 ± 1.6 cm; *Gclm^−/−^*: 35.7 ± 1.6 cm). Swim speed was also the same in wild-type (19.2 ± 0.4 cm/sec) and *Gclm^−/−^* (18.5 ± 0.4 cm/sec) mice.

## 4. Discussion

Genetic deletion of *GCLM* leads to low GSH levels in all tissues including the brain [6, 9–11]. Neurons and astrocytes from *Gclm^−/−^* mice have been shown to be particularly susceptible to the neurotoxic effects of various chemicals, such as domoic acid, polychlorinated biphenyls, methylmercury, certain organophosphorus insecticides, and polybrominated flame retardants, known to elicit oxidative stress [6, 10, 12, 13], and preliminary *in vivo* experiments with the flame retardant BDE-47 (2,2′,4,4′-tetrabromodiphenyl ether) have supported these findings (Giordano and Costa, unpublished). In order to carry out *in vivo* exposure studies with such compounds, to ascertain their potential ability to disrupt normal behavior, a behavioral characterization of *Gclm^−/−^* mice was first needed. 

Results of the present study indicate that *Gclm^−/−^* mice did not differ from wild-type mice in a number of behaviors aimed at testing spontaneous motor activity, motor coordination, learning and memory, and sensory functions. These tests are normally used in behavioral toxicology to assess adverse effects of chemicals on the nervous system upon adult or developmental exposure to chemicals [29]. The lack of differences between *Gclm^−/−^* and wild-type mice in these tests would suggest that GSH deficiency does not lead to alterations in nervous system function significant enough to be detected. However, significant differences would be expected upon challenges with exogenous compounds, particularly those which elicit oxidative stress. As such, *Gclm^−/−^* mice would represent a good model to assess the importance of such gene-environment interactions leading to neurotoxicity. 

Two additional explanations for the lack of differences observed between *Gclm^−/−^* mice and wild-type mice in the present study may lie in the types of behavioral tests utilized and in the age of the animals. Whilst this work was in progress, another study investigating behavioral alterations in *Gclm^−/−^* mice was published [11]. These investigators not only confirmed some of our findings, but also evidenced additional subtle alterations. As in our study, no differences were found in spatial working memory, spatial reference and learning memory, and spatial reversal learning. However, in other tests, *Gclm^−/−^* mice (4–6 month-old) displayed increased novelty-induced exploration, altered behavior during an object recognition task, reduced behavioral inhibition under stress, and lower response to delayed fear conditioning [11]. These subtle behavioral alterations were attributed to oxidative changes in the ventral hippocampus, with no changes in the dorsal hippocampus, and were suggested to be potentially relevant in schizophrenia [11]. However, while an association between *Gclm* polymorphisms and schizophrenia had been previously suggested [16], more recent studies have challenged this hypothesis [17, 18]. 

In addition, it is possible that there are subtle differences between these two *Gclm^−/−^* models, since the strategy for gene deletion was slightly different. In the model first published by Dalton et al. [5] and used by Steullet et al. [11], the first exon of the *Gclm* gene was replaced with a neomycin phosphotransferase (*neo*) in reverse orientation. In the model we have used here, the first exon was replaced with a beta-galactosidase/neomycin phosphotransferase (*β-geo*) fusion gene in the forward orientation. While these two *Gclm^−/−^* models appear to have essentially identical changes in GSH levels, it is still possible that the two different approaches used to construct these models might have resulted in other unforeseen differences in their biology.

Mice tested in this study were young adults, between 13 and 20 weeks of age. It has been proposed that oxidative stress may contribute to aging by progressively increasing oxidant damage to cells [30, 31]. In addition, oxidative stress has been suggested to be involved in neurodegenerative diseases, such as Parkinson's and Alzheimer's diseases and amyotrophic lateral sclerosis [19, 20, 32]. It has been recently shown that fibroblasts from *Gclm^−/−^* mice undergo premature senescence, as evidenced by altered cell morphology, diminished growth rate, and increased senescence-associated *β*-galactosidase activity [33]. Thus, it is plausible that *Gclm^−/−^* would display altered behaviors, compared to their wild-type counterparts, as they age and thus become a useful model for studies on the effects of altered antioxidant capacity on the aging of the nervous system.

## Figures and Tables

**Table 1:** Body weights of adult mice.

	Male	Female
Wild-type	26.70 ± 1.94 g	19.79 ± 1.62 g
*Gclm*-hemizygous	26.16 ± 2.91 g	19.16 ± 1.50 g
*Gclm^−/−^*	*23.92 ± 1.34 g	*17.37 ± 1.04 g

Mice were 12–20-week old. Results represent the mean (±SD) of 16–36 animals.

*Significantly different from the respective wild-type (*P* < .0001).
